# Inhibition of PPARγ during rat pregnancy causes intrauterine growth restriction and attenuation of uterine vasodilation

**DOI:** 10.3389/fphys.2013.00184

**Published:** 2013-07-23

**Authors:** Natalia I. Gokina, Siu-Lung Chan, Abbie C. Chapman, Karen Oppenheimer, Thomas L. Jetton, Marilyn J. Cipolla

**Affiliations:** ^1^Department of Obstetrics, Gynecology and Reproductive Sciences, College of Medicine, University of VermontBurlington, VT, USA; ^2^Department of Neurological Sciences, College of Medicine, University of VermontBurlington, VT, USA; ^3^Department of Medicine, College of Medicine, University of VermontColchester, VT, USA

**Keywords:** uterine and mesenteric artery, pregnancy, PPARγ, vasodilation, preeclampsia

## Abstract

Decreased peroxisome proliferator-activated receptor gamma (PPARγ) activity is thought to have a major role in preeclampsia through abnormal placental development. However, the role of PPARγ in adaptation of the uteroplacental vasculature that may lead to placental hypoperfusion and fetal growth restriction during pregnancy is not known. Here, pregnant Sprague–Dawley rats (*n* = 11/group) were treated during the second half of pregnancy with the PPARγ inhibitor GW9662 (10 mg/kg/day in food) or vehicle. Pregnancy outcome and PPARγ mRNA, vasodilation and structural remodeling were determined in maternal uterine and mesenteric arteries. PPARγ was expressed in uterine vascular tissue of both non-pregnant and pregnant rats with ~2-fold greater expression in radial vs. main uterine arteries. PPARγ mRNA levels were significantly higher in uterine compared to mesenteric arteries. GW9662 treatment during pregnancy did not affect maternal physiology (body weight, glucose, blood pressure), mesenteric artery vasodilation or structural remodeling of uterine and mesenteric vessels. Inhibition of PPARγ for the last 10 days of gestation caused decreased fetal weights on both day 20 and 21 of gestation that was associated with impaired vasodilation of radial uterine arteries in response to acetylcholine and sodium nitroprusside. These results define an essential role of PPARγ in the control of uteroplacental vasodilatory function during pregnancy, an important determinant of blood flow to the placenta and fetus. Strategies that target PPARγ activation in the uterine circulation could have important therapeutic potential in treatment of pregnancies complicated by hypertension, diabetes or preeclampsia.

## Introduction

Preeclampsia, a life-threatening complication of human pregnancy characterized by proteinuria and the new-onset of hypertension after 20 weeks of gestation, is a major cause of maternal and fetal morbidity and mortality worldwide (Roberts, 1998; Alexander et al., 2001; Sibai et al., 2005). Although the etiology of this pregnancy-related disease remains unknown, placental hypoxia/ischemia due to shallow placentation and reduced uterine blood flow is considered an essential event triggering systemic hypertension and intrauterine growth restriction (Roberts et al., 2003; Gilbert et al., 2008). During normal pregnancy, the uterine circulation undergoes considerable structural and functional changes to facilitate a large increase in uterine blood flow necessary for successful pregnancy (Sibai et al., 2005; Osol and Mandala, 2009). However, during preeclampsia, this circulation is significantly affected due to decreased vasodilator production and outward remodeling of uterine spiral vessels (Roberts, 1998; Sibai et al., 2005; Gilbert et al., 2008; Burton et al., 2009). These structural and functional abnormalities in the uteroplacental bed during preeclampsia are likely important for the progression of placental hypoperfusion associated with the condition.

Peroxisome proliferator-activated receptor gamma (PPARγ) is a ligand-activated nuclear hormone receptor that upon activation, heterodimerizes with retinoid-X-receptor. This complex then binds to DNA-specific sequences to promote transcription of target genes (Desvergne and Wahli, 1999; Marx et al., 2004; Desvergne et al., 2006). PPARγ is extensively expressed in white adipose tissue and plays a critical role in the regulation of adipocyte differentiation and function (Desvergne et al., 2006). PPARγ also has an important role in pregnancy. Studies using PPARγ-null mice unexpectedly revealed a vital role of these receptors in placental development. PPARγ-null placentas exhibit abnormal terminal differentiation of the trophoblast and deficient placental vascularization leading to embryonic lethality by mid-gestation. Restoration of PPARγ gene via chimeras resulted in rescue of mutant embryos indicating that PPARγ deficiency is the major cause of fetal death (Barak et al., 1999; Kubota et al., 1999). Recent studies have also demonstrated that PPARγ expression has an essential role in trophoblast differentiation, invasion and metabolism in rodent and human placenta (Asami-Miyagishi et al., 2004; Fournier et al., 2007; Barak et al., 2008; Giaginis et al., 2008).

There is compelling evidence to suggest an association between PPARγ and preeclampsia. Circulating activators of PPARγ are reduced in preeclamptic pregnancy weeks before the onset of maternal symptoms (Waite et al., 2000, 2005). In addition, women with Pro467Leu mutation, a dominant negative mutation of PPARγ, had pregnancies complicated with gestational diabetes and severe preeclampsia (Barroso et al., 1999). Recent studies in rat models also suggest an important role for PPARγ in preeclampsia. Inhibition of PPARγ during pregnancy in rats with the ligand inhibitor T0070907 caused key features of preeclampsia to develop, including elevated mean arterial pressure, proteinuria, systemic endothelial dysfunction, and reduced fetal weight (McCarthy et al., 2011a). In addition, treatment with the PPARγ activator rosiglitazone in a placental ischemia model of preeclampsia ameliorated hypertension in pregnant rats and improved mesenteric artery vasodilation, suggesting PPARγ as a potential therapeutic target in the treatment of preeclampsia (McCarthy et al., 2011b).

PPARγ is also expressed in vascular tissue and shown to have a significant role in the cardiovascular system (Law et al., 2000; Calnek et al., 2003; Kanie et al., 2003; Bagi et al., 2004). Inhibition of PPARγ causes inward remodeling, oxidative stress and endothelial dysfunction of arteries independent of changes in blood pressure (Sigmund, 2010). However, in spite of the well-documented function of PPARγ in placental development and its association with preeclampsia, expression of these receptors and their role in the control of uteroplacental blood flow during pregnancy remains unknown. In this study, we hypothesized that decreased activity of PPARγ impairs maternal uteroplacental remodeling and vasodilation during pregnancy resulting in fetal growth restriction and abnormal placentation. Thus, the objectives of the present study were to (1) characterize the effect of PPARγ inhibition with oral administration of GW9662, a selective ligand inhibitor of PPARγ, on maternal and fetal pregnancy outcome; (2) evaluate endothelial function of uteroplacental and mesenteric resistance arteries under conditions of decreased PPARγ activity during second half of rat pregnancy, and (3) to explore the effect of GW9662 on uterine vascular growth and mechanical properties of uteroplacental and mesenteric arteries.

## METHODS

### Animals

Pregnant (6–8 days) Sprague–Dawley rats (11–12 weeks of age) were purchased from Charles River Laboratories ( St. Constant, QC, Canada) and housed in the animal care facility at the University of Vermont. Animals were randomly selected and treated with either the PPARγ inhibitor GW9662 (10 mg/kg/day for 10 days in food; *n* = 11) or vehicle (*n* = 11). Treatment with GW9662 or vehicle was started at day 10 of pregnancy. The dose of GW9662 (10 mg/kg/day) was higher than has been reported in other studies (3 mg/kg/day; Bagi et al., 2004) due to higher activation of PPARγ in pregnancy (Waite et al., 2000). GW9662 is a highly specific irreversible inhibitor of PPARγ and a valuable tool for determining PPARγ receptor-mediated functions in different biological systems (Leesnitzer et al., 2002). Rats were studied on day 20 (*n* = 8) or 21(*n* = 3) of pregnancy. All experiments were conducted in accordance with the National Institutes of Health *Guide for the Care and Use of Laboratory Animals*, and all protocols were approved by the Institutional Animal Care and Use Committee of the University of Vermont.

### Blood pressure and glucose measurements

Blood pressures (systolic, diastolic and mean) were measured every 2–3 days during treatment by tail cuff as previously described (Chan et al., 2010). Blood glucose levels were determined every other day using a Freestyle glucometer.

### Uterine vascular dimensions and pregnancy outcome

On day 20 (21) of pregnancy, rats were euthanized under anesthesia. The mesentery with vasculature, and the gravid uterus and uterine vasculature were carefully removed and placed in a dissecting dish containing cold, physiologic salt solution (PSS). Morphometric measurements of the unstretched uterine vasculature of both uterine horns were completed as previously described (Phillips et al., 2012). After morphometric measurements, fetuses and their placentas were individually weighed without membranes and umbilical cords. Fetal outcome of day 20 and 21 control and treated pregnant rats was characterized separately as there is a significant difference in fetal and placental weights between day 20 and 21. Also, due to progressive growth of maternal uterine vasculature during the last 5 days of rat pregnancy, all morphometric and structural vascular measurements were performed using vessels from 20 day pregnant rats.

### Radial uterine artery (RUA) and mesenteric artery (MA) vasodilation

In rodent and human pregnancy with hemochorial placentation, RUAs are the major site of uteroplacental vascular resistance (Moll, 2003). Thus, these are key arteries controlling uteroplacental blood flow. Second-order RUAs feeding the placenta (uteroplacental) were carefully dissected and used for *in vitro* experimentation as previously described (Gokina and Goecks, 2006). Uteroplacental radial arteries from late pregnant rats can develop vasoconstriction (myogenic tone) in response to elevations of pressure exceeding 50 mmHg. To avoid development of myogenic tone and its interference with phenylephrine-induced constriction, arteries were pressurized at 50 mmHg. After a 1 h equilibration period and elevation of intraluminal pressure from 10 to 50 mmHg, phenylephrine (PE) was applied in increasing concentrations (1–3 doses) to produce a constriction of 50–70% of the initial diameter. Following stabilization of diameter, acetylcholine (ACh) was added to the bath in increasing concentrations to assess endothelial function. ACh was washed out and the nitric oxide synthase (NOS) inhibitor nitro-L-arginine (L-NNA; 200 μmol/l) was added. Vessels were again pre-constricted with PE and the nitric oxide (NO) donor sodium nitroprusside (SNP) was applied in increasing concentrations to assess smooth muscle responsiveness to NO. Finally, a combination of papaverine (100 μmol/l) and diltiazem (10 μmol/l) was added to obtain fully relaxed diameters and perform passive measurements for assessment of mechanical properties. ACh- or SNP-induced vasodilation was expressed as the percentage of maximal vasodilation in response to papaverine and diltiazem (D_max_). Similar parallel experiments were performed using 3rd order MAs dissected out from control (*n* = 8) and GW9662-treated (*n* = 8) rats.

To characterize the effect of GW9662 on endothelium-derived hyperpolarizing factor (EDHF)-mediated vasodilation, ACh effects were also tested on RUA pre-treated with 200 μmol/l L-NNA and 10 μmol/l indomethacin for 20 min before testing the effects of PE and ACh to inhibit endogenous production of NO and prostacyclin.

### Passive mechanical properties of arteries

Changes in passive mechanical properties (distensibility, stress-strain relationships) and remodeling (inner and outer diameters, wall thickness) were measured for arteries from 20 day pregnant rats under fully relaxed conditions, as previously described (Phillips et al., 2012). Radial uterine arteries (RUA) were given papaverine (100 μmol/l) and diltiazem (10 μmol/l) to fully relax smooth muscle and obtain passive measurements. Lumen diameter changes in response to stepwise elevation in intraluminal pressure from 5 to 125 mmHg were monitored with the SoftEdge Acquisition System (IonOptix, Milton, MA). Arterial wall thickness (h) was measured from images of pressurized arteries after stabilization of the lumen diameter (D) at each specific level of pressure. For the calculation of circumferential wall stress, intraluminal pressure was converted from mmHg to N/m^2^ (1 mmHg = 1.334 × 10^2^ N/m^2^). Circumferential stress, (σ) was calculated using the equation: σ = PxD/2h. Circumferential strain (ε) was calculated according to the following equation: ε = (D − D_5_)/D_5_, where D_5_ represents the lumen arterial diameter at the lowest (5 mmHg) intraluminal pressure. The rate constant of an exponential function fitted to stress-strain curve (stiffness coefficient β) for each artery was determined using SigmaPlot software program and used to compare stress-strain relationships between two groups of arteries.

Similarly, changes in passive arterial diameters and vessel wall thickness in response to stepwise elevation in intraluminal pressure were measured from fully relaxed 3rd order mesenteric arteries of control and GW9662-treated rats.

### Tissue collection and quantitative RT PCR

RUAs and main uterine arteries were collected from non-pregnant (*n* = 6) and untreated late-pregnant (*n* = 7) rats for real-time PCR analysis of PPARγ expression. In addition, expression of PPARγ was compared in main uterine arteries and second order MAs from 20 day pregnant rats (*n* = 3) as well as in main uterine arteries of GW9662- and vehicle-treated rats. Placentas were collected from control and treated rats to determine the expression of soluble fms-like tyrosine kinase 1(sFlt-1) mRNA. Visceral mesenteric fat was collected from vehicle- and GW9662-treated late pregnant rats (*n* = 11) for real-time PCR analysis of leptin expression to assess treatment efficiency. All PCR was performed as previously described (Chan et al., 2010). RNA was extracted from arteries utilizing a TRIzol (Invitrogen) extraction protocol and further purified using a RNeasy Micro Kit (Qiagen). All samples were quantified using a Nanodrop Spectrophotometer and RNA integrity was assessed on the Agilent 2100 Bioanalyzer (Agilent Technologies). Fifty ng of total RNA was reverse transcribed using the iScript cDNA Synthesis Kit (Biorad). QPCR reactions were done using 150 nM of forward and reverse primers in Power Sybrgreen Master Mix (Applied Biosystems) with 1 μl of cDNA template. Target mRNA transcripts for PPARγ, leptin and two housekeeping genes (Hprt and Ywhaz) were amplified using an ABI Prism 7000 Sequence Detection System. Relative quantification was determined using the comparative Ct method (2^−ΔΔCT^). Standard curves were generated for each primer set to ensure that primer efficiencies were within 10% of each other to allow for this analysis choice. Relative target mRNA values were normalized using the mean of the control gene quantities and calibrated to a control sample in each group. Negative water controls were run for each primer set to ensure no contamination in the reagents and that no secondary primer structures were amplified. In each primer set at least one primer was designed over an exon-exon junction. We used the following primers: PPARγ_F (GTCTCACAATGCCATCAGGTTT); PPARγ_R (TCAGCGGGAAGGACTTTATGTAT); Hprt_F (CAGTCCCAGCGTCGTGAT); Hprt_R (CAAGTCTTTCAGTCCTGTCCATAA); Ywhaz_F (GCAACGACGTACTGTCTCTTTTGG); Ywhaz_R (GTCCACAATTCCTTTCTTGTCATC); Leptin F (TTTCACACACGCAGTCGvGTATCC); Leptin R (AGATGGAGGAGGTCTCGCAG).

sFlt-1 F (TGTGGCACCCCTGTCACTACA); sFlt-1 R (ACCGTCTTATTGGTTCCTTCTATGACC).

### Uterine vascular dimensions

A stereomicroscope (Zeiss Stemi 2000-C) with a calibrated reticule was used to determine the length of the main uterine artery, the distance between the main uterine artery and placenta or myometrium in the center of each uterine horn.

### Solutions and drugs

The PSS used for isolated artery experiments contained (mmol/l): 119 NaCl, 4.7 KCl, 24.0 NaHCO_3_, 1.2 KH_2_PO_4_, 1.6 CaCl_2_, 1.2 MgSO_4_, 0.023 EDTA, and 11.0 glucose, pH = 7.4 (maintained by aeration with 5% O_2_, 10% CO_2_ and 85% N_2_). All chemicals were purchased from Sigma Chemical Co. (St. Louis, MO). GW9662 was purchased from Cayman Chemical (Ann Arbor, MI, USA). Diltiazem was prepared as a 10 mmol/L stock solution in deionized water and kept (1–2 weeks) refrigerated until use. Indomethacin was prepared in ethanol and L-NNA was dissolved in PSS. ACh, PE, SNP and papaverine were dissolved in deionized water, prepared and used on the day of the experiment.

### Statistical analysis

Arterial diameter and pressure were simultaneously recorded with an IonOptix data acquisition program and imported into SigmaPlot program for graphical representation, calculations, and statistical analysis. Because of oscillatory pattern of the responses in arterial diameter, we calculated a mean value for diameters that represents average data points over period of 15–20 s obtained using IonOptix software, and includes 1–3 oscillations when present. An unpaired Student's *t*-test or Two-Way repeated measures ANOVA with a Holm-Sidak *post-hoc* test (SigmaPlot software) was used to compared groups. Significant differences were defined as *p* < 0.05. Data are presented as mean ± SEM, where *n* is the number of arterial segments studied. One artery per animal was used for reactivity and structural measurements.

## Results

### Maternal and fetal outcome

GW9662 treatment of pregnant rats did not affect maternal blood glucose levels, body weight or blood pressure (Table 1). Oral administration of GW9662 during the second half of pregnancy resulted in significantly reduced fetal weights on day 20 (2.21 ± 0.02 g vs. 2.30 ± 0.02 g; *p* < 0.05) and 21 (3.40 ± 0.07 g vs. 3.75 ± 0.03 g; *p* < 0.01) of pregnancy. Placental weights were similar between control and GW9662-treated rats on 20 day of pregnancy (0.45 ± 0.01 g vs. 0.44 ± 0.01 g; *p* > 0.05) but were significantly increased in the treated group on day 21 of pregnancy (0.54 ± 0.02 g vs. 0.48 ± 0.01 g; *p* < 0.01). There were no significant differences in the number of fetal resorptions (0.3 ± 0.2 vs. 0.8 ± 0.4; *p* > 0.05) or litter size (14.0 ± 0.6 vs. 14.2 ± 0.9; *p* > 0.05) between GW9662-treated and control groups, respectively (Table 1).

**Table 1:** **Maternal physiological parameters and pregnancy outcome of Sprague–Dawley rats**.

**Animal**	**Maternal weight, (g)**	**Blood glucose (mg/dL)**	**BP systolic, (mmHg)**	**BP diastolic, (mmHg)**	**BP Mean (mmHg)**	**Litter size (number of pups)**	**Number of resorptions**
Untreated (*n* = 11)	377 ± 9	87 ± 4	117 ± 6	87 ± 5	95 ± 5	14 ± 0.9	0.8 ± 0.4
GW9662-treated (*n* = 11)	387 ± 6	88 ± 4	116 ± 4	86 ± 4	96 ± 4	14 ± 0.6	0.3 ± 0.2

### PPARγ expression in uterine and mesenteric arteries

**Figure 1:**
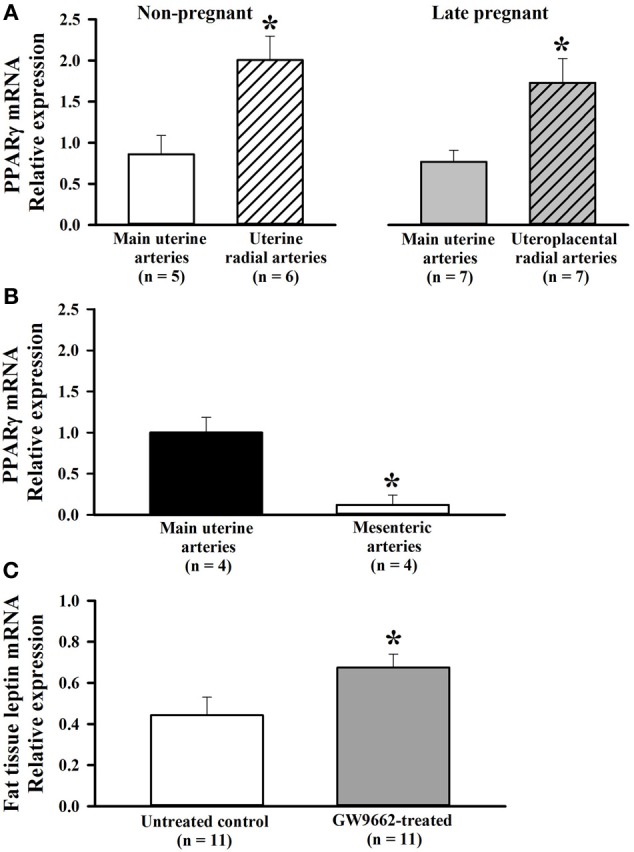
**(A)** Bar graphs showing relative expression of PPARγ in uterine arteries of non-pregnant and pregnant rats. PPARγ mRNA was detected in both types of uterine arteries. A comparison between main and radial uterine arteries demonstrates significantly greater PPARγ expression in smaller radial resistance arteries of non-pregnant and late pregnant rats. mRNA is expressed relative to the main uterine artery sample from a control rat. ^*^p < 0.05 vs. main uterine artery. (**B)** Higher expression of PPARγ in uterine compared to mesenteric vasculature of late pregnant rats. Quantitative RT-PCR results showing relative expression of PPARγ mRNA in mesenteric and main uterine arteries obtained from the same rats (*n* = 4). Results analyzed using the ddct method and normalized to Ywhaz. mRNA is expressed relative to the sample from main uterine artery. **(C)** Bar graph showing relative leptin mRNA expression, a PPARγ target gene, in adipose tissue from control and GW9662-treated pregnant rats. GW9662 treatment caused a significant increase in leptin mRNA, demonstrating that treatment to inhibit PPARγ was effective. ^*^p < 0.05 vs. control.

PPARγ is expressed in vascular cells and shown to have a role in regulating vascular structure and function (Sigmund, 2010). However, if PPARγ is expressed in uterine vasculature and if PPARγ expression is modulated during pregnancy has not been shown. Thus, we compared relative mRNA expression of PPARγ in main uterine and RUAs from non-pregnant (*n* = 6) and late-pregnant rats (*n* = 7) using quantitative PCR methodology. As shown in Figure 1A, PPARγ was expressed in both types of uterine arteries, with a significantly greater expression in the smaller RUAs compared to larger main uterine arteries. Pregnancy did not modulate the expression of the PPARγ in both types of vessels. When compared to vessels from a non-reproductive organ, relative PPARγ mRNA levels were significantly higher in main uterine artery compared to MAs of late-pregnant rats (Figure 1B).

### Placental sFlt-1 expression

sFlt-1 mRNA levels were determined in the placentas from control (*n* = 7) and GW9662-treated (*n* = 8) rats. There was no significant increase in total sFlt-1 expression in GW9662-treated rats compared to controls (0.81 ± 0.11 vs. 0.74 ± 0.08 relative to a sample from a control rat).

### Leptin expression in adipose tissue with GW9662 treatment

To ascertain that treatment with GW9662 was effective at inhibiting PPARγ, leptin mRNA expression was measured in adipose tissue where both PPARγ and leptin are highly expressed. Leptin is a well-known PPARγ target gene (Desvergne et al., 2006). In PPARγ^+/−^ mice where the activity of the receptors is moderately reduced, leptin mRNA in white adipose tissue and circulating levels of leptin were higher compared to wild type animals (Kubota et al., 1999). On the other hand, PPARγ activators reduced the expression of leptin receptors in adipose tissue (Kallen and Lazar, 1996). Based on these observations, leptin expression was selected as an indicator of PPARγ activity in this study. Figure 1C shows that leptin expression was significantly increased with GW9662 treatment compared to vehicle, demonstrating that inhibition of PPARγ during the last half of pregnancy was effective at altering PPARγ-specific gene expression. Thus, treatment with GW9662 appeared to inhibit PPARγ as expected.

### GW9662 effect on RUA reactivity

**Figure 2:**
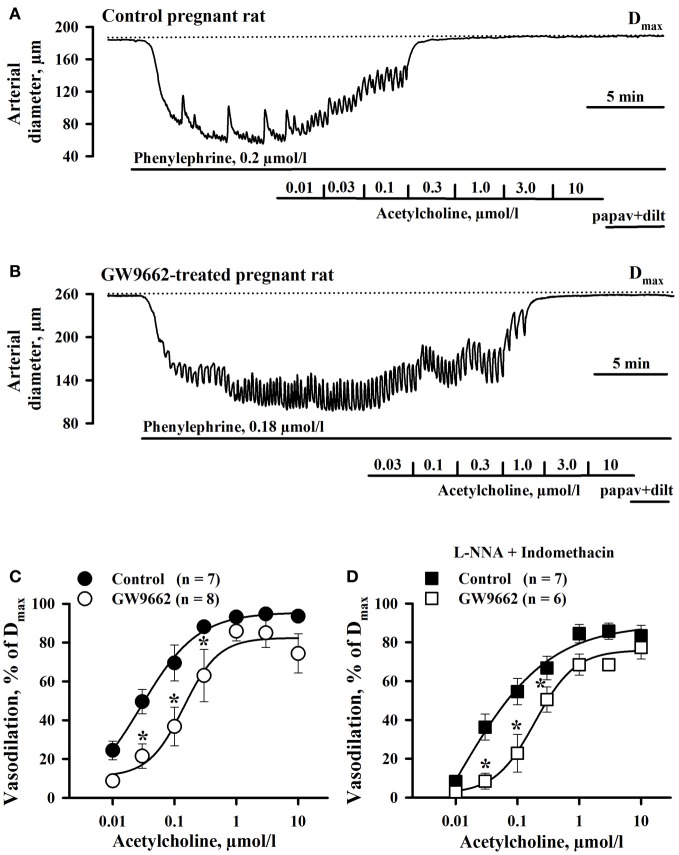
**Chronic inhibition of PPARγ during the second half of rat pregnancy resulted in impairment of uterine vasodilation**. Representative changes in the arterial diameter of radial uteroplacental arteries from a control **(A)** and a GW9662-treated **(B)** pregnant rats induced by cumulative application of ACh. The arteries were pre-constricted with phenylephrine before testing ACh. Dotted lines show maximal diameters (D_max_) of the arteries dilated in a relaxing solution containing 10 μmol/l diltiazem and 100 μmol/l papaverine. **(C)** Graph summarizing the effect of oral administration of GW9662 during pregnancy on concentration-dependent dilation of uteroplacental arteries to ACh; **(D)** Graph showing attenuation of EDHF-mediated uterine vasodilation induced by GW9662 treatment. The arteries were pre-treated for 20 min with 200 μmol/l L-NNA and 10 μmol/l indomethacin before testing ACh. ^*^p < 0.05 vs. control.

The effect of PPARγ inhibition on endothelial function of RUAs was determined as these vessels are the site of vascular resistance in the uteroplacental bed and thus an effect on their vasodilator properties could influence uterine blood flow. The vasodilator effect of ACh was compared between RUAs from control and GW9662-treated animals after pre-constricting with PE by 63 ± 4% (Control, *n* = 7) and 57 ± 2% (GW9662, *n* = 8) of their initial diameters. Initial diameters of RUAs of control (187.7 ± 6.5 μm) and GW9662-treated (177.3 ± 13.8 μm) rats were not significantly different from their passive diameters measured in the presence of papaverine and diltiazem (192.8 ± 5.1 μm and 177.4 ± 13.4 μm, in RUAs of control and treated rats, respectively). Figures 2A,B show representative diameter changes of arteries from control and GW9662-treated rats in response to cumulative application of ACh. These data, summarized in Figure 2C, demonstrate that inhibition of PPARγ with GW9662 decreased ACh vasodilation of RUA.

We also characterized the effect of GW9662 treatment on NO- and prostacyclin independent (EDHF-mediated) vasodilation of RUA. Figure 2D demonstrates a significant attenuation of EDHF-mediated vasodilation to ACh in vessels from rats pre-treated with GW9662 in the presence of L-NNA and indomethacin.

**Figure 3:**
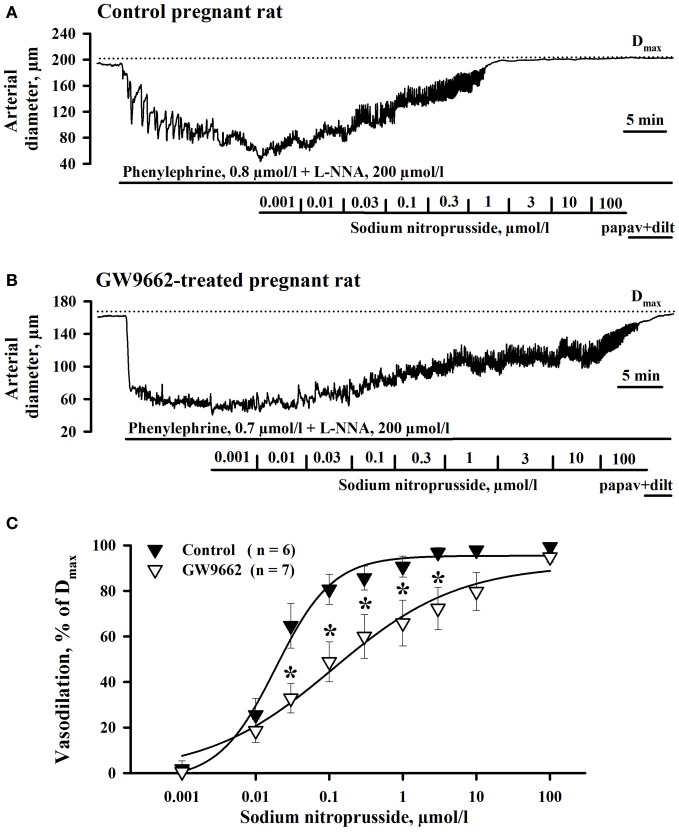
**Attenuation of sodium nitroprusside (SNP)-induced vasodilation of radial uteroplacental arteries by PPARγ inhibition during rat pregnancy**. Representative tracings showing dilatation of uteroplacental arteries from control **(A)** and GW9662-treated **(B)** pregnant rats induced by a cumulative application of SNP. Dotted lines indicate the maximal arterial diameters (D_max_) in the presence of papaverine and diltiazem. **(C)** Graph summarizing the effect of oral administration of GW9662 during pregnancy on concentration-dependent dilation of uteroplacental arteries to SNP. ^*^p < 0.05 vs. control.

Because reactivity of vascular smooth muscle cells (SMCs) to NO might be decreased by exposure of rats to GW9662, we compared the response of RUAs to the NO donor SNP. PPARγ controls NO production in vascular endothelium (Kleinhenz et al., 2009). Chronic exposure of pregnant rats to GW9662 may modulate basal NO generation with consequent change in sensitivity of soluble guanylate cyclase (sGC) to NO in SMCs of uterine arteries (Moncada et al., 1991). To eliminate the differences in NO sensitivity of sGC in control and treated vessels and to determine whether mechanisms of NO-induced SMC relaxation is modulated by GW9662 treatment, endogenous NO production was inhibited with L-NNA. Figures 3A,B show representative diameter changes of arteries from control and GW9662-treated rats in response to cumulative application of SNP. A summary graph on Figure 3C demonstrates that vasodilation to SNP was significantly impaired by GW9662 treatment.

### Maternal uterine vascular remodeling

To characterize the effect of PPARγ inhibition on the growth of the maternal uterine vasculature, measurements of the mesometrial uterine arcade were taken from both uterine horns of 20 day pregnant GW9692-treated (*n* = 8) or control (*n* = 8) rats immediately after euthanasia as previously described (Phillips et al., 2012). There were no differences in the length of the main uterine artery (79.7 ± 3.4 vs. 80.6 ± 2.0 mm) or the distance from the main uterine artery to the placenta (12.3 ± 0.5 vs. 12.2 ± 0.5 mm) or to the myometrium (14.6 ± 0.6 vs. 14.9 ± 0.6 mm) between control and GW9662-treated rats, respectively.

### Effect of GW9662 treatment on passive mechanical properties of uteroplacental arteries

**Figure 4:**
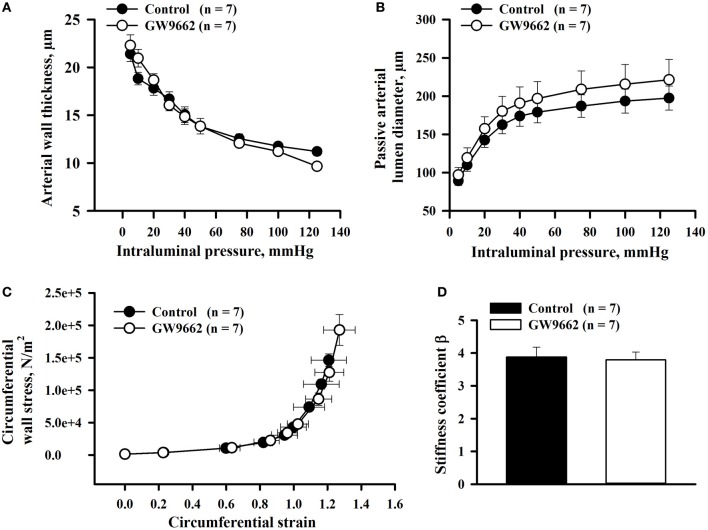
**Passive mechanical properties of radial uteroplacental arteries from control and GW9662-treated rats**. **(A,B)** Arterial wall thickness and passive arterial diameters as a function of intraluminal pressure. **(C,D)** Stress-strain relationship and stiffness coefficients were determined for radial uteroplacental arteries from control and GW9662-treated rats. All measurements were taken from arteries of 20 day pregnant rats.

Arterial wall thickness and passive lumen diameters, indicators of circumferential vascular remodeling, were measured from RUAs of 20 day pregnant rats pressurized at 5–125 mmHg. Despite decreases in vasodilatory responses, there was no effect of GW9662 treatment on wall thickness or passive lumen diameters (Figures 4A,B). Passive distensibility of uteroplacental arteries from GW9662-treated and control animals was also not significantly different between groups (not shown).

Finally, the effect of GW9662 administration during pregnancy on passive mechanical properties of uteroplacental arteries was characterized by the stress-strain relationship and comparing the stiffness coefficient β for each artery. There were no changes in the stress-strain relationship or stiffness coefficient β in RUAs of GW9662-treated vs. control rats (Figures 4C,D).

### GW9662 effect on reactivity and structure of MA

**Figure 5:**
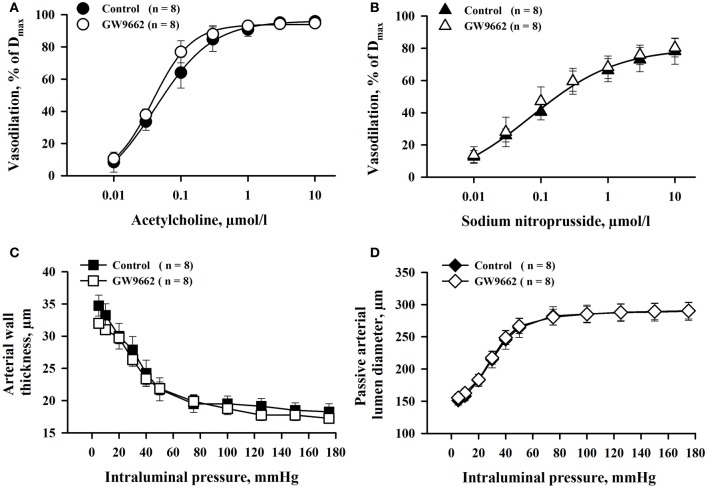
**Chronic inhibition of PPARγ with GW9662 during the second half of rat pregnancy produced no significant changes in ACh- and SNP-induced vasodilation (A,B), and passive mechanical properties (C,D) of mesenteric resistance arteries**. Arteries were pre-constricted with phenylephrine before testing ACh or SNP. Vasodilation is expressed as a percentage of maximal response to application of papaverine and diltiazem (D_max_).

Effect of GW9662 treatment on MA responses to ACh and SNP was determined in parallel experiments. There were no significant differences in ACh- and SNP-induced vasodilation between two groups of vessels (Figures 5A,B). Similarly, GW9662 treatment did not affect passive lumen diameters and wall thickness of MA (Figures 5C,D). Thus, the effect of PPARγ inhibition on vasodilation was specific for RUAs.

## Discussion

This study investigated the role of PPARγ in uteroplacental vascular adaptation to pregnancy and fetal growth by inhibiting PPARγ during the second half of rat pregnancy and assessing fetal outcome, uterine vascular remodeling and vasodilatory function. We found that PPARγ was highly expressed in the uterine circulation and that expression of PPARγ was ~2-fold greater in radial vs. main uterine arteries, suggesting a more prominent role of PPARγ in smaller resistance arteries vs. larger conduit vessels. Inhibition of PPARγ with administration of GW9662 during rat pregnancy resulted in fetal growth restriction and decreased vasodilation of uterine resistance arteries to both endothelium-dependent (ACh) and –independent (SNP) agents. However, growth of the maternal uterine vasculature and passive mechanical properties were not modified by GW9662 administration at this time point in gestation. Together, these results suggest that PPARγ inhibition during the last half of pregnancy selectively affected uterine vascular vasodilatory function without affecting structural remodeling. The association of PPARγ inhibition with fetal growth restriction could implicate vasodilation of the uteroplacental arteries over remodeling as a key mechanism controlling fetal weight during the last half of gestation. In addition, these results implicate PPARγ activation as an important mechanism regulating uterine artery vasodilatory function during pregnancy.

In the present study, a short-term treatment of pregnant rats with GW9662 resulted in significant reduction in fetal weights on days 20 and 21 of pregnancy. The association between decreased PPARγ activation or expression and fetal growth restriction has been demonstrated in several conditions. Recent data indicate that circulating activators of PPARγ are reduced in human pregnancy complicated with preeclampsia that is often associated with intrauterine growth restriction (IUGR) (Wieser et al., 2008). PPARγ expression was found to be diminished in human placentas of small for gestational age fetuses (Diaz et al., 2012). In addition, maternal glucocorticoid exposure results in a marked reduction in PPARγ expression in the labyrinth zone of rat placenta that is also associated with restricted fetal growth (Hewitt et al., 2006). Recently, reduced fetal weight similar to what has been reported in the present study, was also found in pregnant rats treated with T0070907, another PPARγ antagonist (McCarthy et al., 2011a). Collectively these findings suggest an essential link between activity of PPARγ and fetal growth in animal and human pregnancy.

Reduced uterine blood flow during pregnancy is associated with fetal growth restriction (Zimmermann et al., 1997; Alexander et al., 2001; Isler et al., 2003; Sibai et al., 2005). It is well-documented that intrauterine growth restriction caused by hypertensive pregnancy, preeclampsia or high altitude hypoxia, is associated with endothelial dysfunction of maternal uterine arteries (Mateev et al., 2003; Wareing et al., 2005; Kusinski et al., 2012). Treatment with sildenafil improves both maternal uterine vasodilation and fetal growth in a mouse model of preeclampsia (Stanley et al., 2012). These data strongly support impaired uteroplacental vasodilation as a cause of reduced placental perfusion and intrauterine growth restriction in animal and human pregnancies.

Impaired dilatation of the maternal uterine vasculature may be one of the underlying mechanisms of abnormal fetal growth in our study. Indeed, RUAs from GW9662-treated animals were less responsive to both the endothelium-dependent vasodilator ACh and the endothelium-independent vasodilator SNP, suggesting an effect of PPARγ on endothelial and SMC function. Despite decreased vasodilator function with GW9662, there was no effect of PPARγ on uterine vascular remodeling suggesting uterine vasodilation as an important mechanism in regulating uteroplacental blood flow and fetal growth.

Recent studies demonstrate that PPARγ plays a pivotal role in regulating vascular structure in other circulations (Bishop-Bailey, 2000; Marx and Walcher, 2007; Nicolakakis and Hamel, 2010; Sigmund, 2010). In male mice, a dominant-negative mutation in PPARγ resulted in hypertrophy and inward remodeling in cerebral arterioles (Beyer et al., 2008). In addition, a previous study from our lab found that GW9662 treatment resulted in vessel wall hypertrophy of cerebral arteries of non-pregnant and pregnant females (Chan et al., 2010). In the present study, similar exposure of pregnant rats to GW9662 induced no significant change in vascular structure of uterine vessels, suggesting a differential effect of PPARγ in uterine vs. cerebral vascular beds during pregnancy. This is not surprising considering that the uterine vasculature, in contrast to cerebral vessels, is markedly enlarged over the course of pregnancy that may offset the negative effects of decreased PPARγ activity on their structure. Alternatively, the mechanisms that regulate uterine artery expansion during pregnancy may not be under control of PPARγ as in other vascular beds.

To our knowledge, this is the first study to show that PPARγ mRNA is present in the wall of maternal non-pregnant and pregnant uterine vasculature. PPARγ appears to regulate vascular function of uterine arteries during pregnancy since its inhibition results in impaired vasodilatory capacity. Endothelium-dependent and endothelium-independent uterine vasodilation was reduced indicating that both endothelial and SMC function is impaired in pregnant rats chronically exposed to PPARγ inhibitor GW9662. PPARγ are expressed in vascular endothelium where they control expression of multiple genes regulating cell adhesion, oxidative stress, PKC and NO production (Sigmund, 2010). Previously, we have shown that EDHF importantly contributes to uterine vasodilation and is mediated by Ca^2+^-dependent activation of endothelial intermediate and small conductance potassium channels (Gokina et al., 2010). In this study, we demonstrated that NO- and prostacyclin-independent uterine vasodilation was reduced by GW9662 treatment. Exact causes and mechanisms of impaired EDHF-mediated response by chronic inhibition of PPARγ in our study remain unknown and may be induced by increased generation of peroxynitrite (Sharma et al., 2013). Inhibitory effect of peroxynitrite on EDHF-mediated coronary vasodilation was recently demonstrated (Liu et al., 2006).

It has been shown that disruption of endothelial PPARγ resulted in endothelial dysfunction due to impaired production of NO and induction of oxidative stress (Kleinhenz et al., 2009). These mechanisms may also contribute to reduced ACh-induced vasodilation in our study. Inhibition of oxidative stress is one of the key mechanisms of PPARγ protective effects in the vascular wall (Bishop-Bailey, 2000; Bagi et al., 2004; Marx and Walcher, 2007; Matsumoto et al., 2008; Ketsawatsomkron et al., 2010; Sigmund, 2010; McCarthy et al., 2011b). Cerebrovascular dysfunction in mice with a dominant negative mutation of PPARγ was reversed by the superoxide scavenger tempol (Ketsawatsomkron et al., 2010).

Endothelium-independent SNP-induced vasodilation was significantly attenuated by chronic exposure of pregnant rats to GW9662 that suggests impairment of mechanisms mediating NO-induced smooth muscle relaxation in the maternal uterine vasculature. PPARγ are expressed in vascular SMCs, and interference with PPARγ in SMCs resulted in systemic hypertension in part due to attenuation of endothelium-dependent and independent vasodilation (Halabi et al., 2008). Dominant negative mutation of PPARγ in smooth muscle resulted in enhanced myogenic tone through increased activation of PKC and PKC-dependent inhibition of large conductance Ca^2+^-activated potassium channels (BK_Ca_) in mice resistance vasculature (Ketsawatsomkron et al., 2012). BK_Ca_ channels are important target of NO-cGMP-PKG signaling cascade in vascular smooth muscle. Whether dysfunction of BK_Ca_ channels underlies reduced NO-mediated uterine vasodilation in our study remains to be determined.

Finally, due to systemic application of GW9662, indirect effects of PPARγ inhibition in adipose or placental tissues on uterine vascular function cannot be excluded. In the future studies it seems important to define the mechanisms and pathways involved in vasodilation controlled by PPARγ in the maternal uteroplacental circulation.

We were unable to detect any significant changes in maternal weight, blood pressure or glucose levels in response to administration of GW9662 during pregnancy, suggesting relatively modest systemic effects of PPARγ inhibition in this study. This conclusion is also confirmed by the absence of any changes in reactivity and structure of mesenteric arteries of GW9662-treated rats. Low expression of PPARγ in these vessels compared to uterine vasculature in part may explain the lack of significant changes in response to treatment of pregnant rats with GW9662 in our study. In this regard, a recent study showed that administration of the PPARγ antagonist T0070907 to pregnant rats using an intraperitoneal osmotic mini-pump was associated with a more striking (by ~25% vs. ~10% in our study) reduction in fetal weight, a modest elevation in blood pressure and reduced sensitivity of mesenteric arteries to bradykinin (McCarthy et al., 2011a). This endothelial dysfunction was reproduced by overnight incubation of mesenteric arteries from healthy pregnant rats with plasma obtained from T0070907-treated rats. It was suggested that elevated circulating plasma sFlt-1 might be responsible for decreased vasodilation to bradykinin. Placental of sFlt-1mRNA and protein were elevated in T0070907-treated rats implicating the placenta as the main contributor to the increased circulating levels sFlt-1. Therefore, endothelial dysfunction of maternal mesenteric arteries results from rather indirect effect of PPARγ inhibition in this study (McCarthy et al., 2011a). In GW9662-treated rats, placental expression of sFlt-1 was only slightly but not significantly elevated indicating that this treatment was relatively modest compared to that described in experiments with administration of T0070907. This may explain the lack of significant changes in mesenteric artery vasodilation and consequently, lack of changes in systemic blood pressure of GW9662-treated rats in our study.

Although we did not find systemic effects of GW9662 administration, vasodilator function of uteroplacental arteries was significantly impaired. It is worth noting that this is the first study that we are aware of to show an effect of PPARγ inhibition during pregnancy on uterine arteries and thus provides evidence for a vascular mechanism by which PPARγ may influence fetal growth. These results also suggest that compromised uteroplacental blood flow may be an early consequence of decreased activity of PPARγ during pregnancy. In addition, decreased maturation of trophoblasts in response to PPARγ inhibition (McCarthy et al., 2011a) may result in diminished remodeling of spiral arteries and contribute to reduced placental blood flow and fetal growth restriction found in our study. Under conditions of more severe reduction in PPARγ activity, we speculate that a greater decrease in uteroplacental blood flow may result in placental ischemia followed by increased circulating anti-angiogenic factors and endothelial dysfunction similar to that found in the recent study of McCarthy et al. (2011a).

The results of the current study indicate that PPARγ plays an essential role in the control of uteroplacental vasodilatory function during pregnancy, an important determinant of blood flow to the placenta and fetus during pregnancy. Our data uncover uterine vascular dysfunction as an early mechanism that may lead to placental hypoperfusion and intrauterine growth restriction in human pregnancies associated with negative mutations in PPARγ or reduced levels of circulating PPARγ activators, such as that seen in preeclampsia (Barroso et al., 1999; Wieser et al., 2008). The use of natural [e.g., relaxin (Chan and Cipolla, 2011)] or synthetic PPARγ activators, or strategies directed to increase PPARγ expression or activity in the uterine vasculature may have important therapeutic potential in treatment of pregnancies complicated by hypertension, diabetes or preeclampsia.

### Conflict of interest statement

The authors declare that the research was conducted in the absence of any commercial or financial relationships that could be construed as a potential conflict of interest.

## References

[B1] Alexander B. T. Bennett W. A. Khalil R. A. Granger J. P. (2001). Preeclampsia: linking placental ischemia with cardiovascular-renal dysfunction. News Physiol. Sci. 16, 282–286 11719606 10.1152/physiologyonline.2001.16.6.282

[B2] Asami-Miyagishi R. Iseki S. Usui M. Uchida K. Kubo H. Morita I. (2004). Expression and function of PPARgamma in rat placental development. Biochem. Biophys. Res. Commun. 315, 497–501 10.1016/j.bbrc.2004.01.074 14766236

[B3] Bagi Z. Koller A. Kaley G. (2004). PPARgamma activation, by reducing oxidative stress, increases NO bioavailability in coronary arterioles of mice with Type 2 diabetes. Am. J. Physiol. Heart Circ. Physiol. 286, H742–H748 10.1152/ajpheart.00718.2003 14551045

[B4] Barak Y. Nelson M. C. Ong E. S. Jones Y. Z. Ruiz-Lozano P. Chien K. R. (1999). PPAR gamma is required for placental, cardiac, and adipose tissue development. Mol. Cell 4, 585–595 10.1016/S1097-2765(00)80209-9 10549290

[B5] Barak Y. Sadovsky Y. Shalom-Barak T. (2008). PPAR signaling in placental development and function. PPAR Res. 2008, 142082 10.1155/2008/142082 18288278 PMC2225458

[B6] Barroso I. Gurnell M. Crowley V. E. Agostini M. Schwabe J. W. Soos M. A. (1999). Dominant negative mutations in human PPARgamma associated with severe insulin resistance, diabetes mellitus and hypertension. Nature 402, 880–883 10622252 10.1038/47254

[B7] Beyer A. M. Baumbach G. L. Halabi C. M. Modrick M. L. Lynch C. M. Gerhold T. D. (2008). Interference with PPARgamma signaling causes cerebral vascular dysfunction, hypertrophy, and remodeling. Hypertension 51, 867–871 10.1161/HYPERTENSIONAHA.107.103648 18285614 PMC2408877

[B8] Bishop-Bailey D. (2000). Peroxisome proliferator-activated receptors in the cardiovascular system. Br. J. Pharmacol. 129, 823–834 10.1038/sj.bjp.0703149 10696077 PMC1571927

[B9] Burton G. J. Woods A. W. Jauniaux E. Kingdom J. C. (2009). Rheological and physiological consequences of conversion of the maternal spiral arteries for uteroplacental blood flow during human pregnancy. Placenta 30, 473–482 10.1016/j.placenta.2009.02.009 19375795 PMC2697319

[B10] Calnek D. S. Mazzella L. Roser S. Roman J. Hart C. M. (2003). Peroxisome proliferator-activated receptor gamma ligands increase release of nitric oxide from endothelial cells. Arterioscler. Thromb. Vasc. Biol. 23, 52–57 10.1161/01.ATV.0000044461.01844.C9 12524224

[B12] Chan S. L. Chapman A. C. Sweet J. G. Gokina N. I. Cipolla M. J. (2010). Effect of PPARgamma inhibition during pregnancy on posterior cerebral artery function and structure. Front. Physiol. 1:130 10.3389/fphys.2010.00130 21423372 PMC3059960

[B11] Chan S. L. Cipolla M. J. (2011). Relaxin causes selective outward remodeling of brain parenchymal arterioles via activation of peroxisome proliferator-activated receptor-gamma. FASEB J. 25, 3229–3239 10.1096/fj.10-175471 21602449 PMC3157692

[B13] Desvergne B. Michalik L. Wahli W. (2006). Transcriptional regulation of metabolism. Physiol. Rev. 86, 465–514 10.1152/physrev.00025.2005 16601267

[B14] Desvergne B. Wahli W. (1999). Peroxisome proliferator-activated receptors: nuclear control of metabolism. Endocr. Rev. 20, 649–688 10.1210/er.20.5.649 10529898

[B15] Diaz M. Bassols J. Lopez-Bermejo A. Gomez-Roig M. D. de Zegher F. Ibanez L. (2012). Placental expression of peroxisome proliferator-activated receptor gamma (PPARgamma): relation to placental and fetal growth. J. Clin. Endocrinol. Metab. 97, E1468–E1472 10.1210/jc.2012-1064 22689692

[B16] Fournier T. Tsatsaris V. Handschuh K. Evain-Brion D. (2007). PPARs and the placenta. Placenta 28, 65–76 10.1016/j.placenta.2006.04.009 16834993

[B17] Giaginis C. Spanopoulou E. Theocharis S. (2008). PPAR-gamma signaling pathway in placental development and function: a potential therapeutic target in the treatment of gestational diseases. Expert Opin. Ther. Targets 12, 1049–1063 10.1517/14728222.12.8.1049 18620525

[B18] Gilbert J. S. Ryan M. J. LaMarca B. B. Sedeek M. Murphy S. R. Granger J. P. (2008). Pathophysiology of hypertension during preeclampsia: linking placental ischemia with endothelial dysfunction. Am. J. Physiol. Heart Circ. Physiol. 294, H541–H550 10.1152/ajpheart.01113.2007 18055511

[B19] Gokina N. I. Goecks T. (2006). Upregulation of endothelial cell Ca^2+^ signaling contributes to pregnancy-enhanced vasodilation of rat uteroplacental arteries. Am. J. Physiol. Heart Circ. Physiol. 290, H2124–H2135 10.1152/ajpheart.00813.2005 16327017

[B20] Gokina N. I. Kuzina O. Y. Vance A. M. (2010). Augmented EDHF signaling in rat uteroplacental vasculature during late pregnancy. Am. J. Physiol. Heart Circ. Physiol. 299, H1642–H1652 10.1152/ajpheart.00227.2010 20817830 PMC2993200

[B21] Halabi C. M. Beyer A. M. de Lange W. J. Keen H. L. Baumbach G. L. Faraci F. M. (2008). Interference with PPAR gamma function in smooth muscle causes vascular dysfunction and hypertension. Cell Metab. 7, 215–226 10.1016/j.cmet.2007.12.008 18316027 PMC2275166

[B22] Hewitt D. P. Mark P. J. Waddell B. J. (2006). Placental expression of peroxisome proliferator-activated receptors in rat pregnancy and the effect of increased glucocorticoid exposure. Biol. Reprod. 74, 23–28 10.1095/biolreprod.105.045914 16135695

[B23] Isler C. M. Bennett W. A. Rinewalt A. N. Cockrell K. L. Martin J. N. Jr. Morrison J. C. (2003). Evaluation of a rat model of preeclampsia for HELLP syndrome characteristics. J. Soc. Gynecol. Investig. 10, 151–153 10.1016/S1071-5576(03)00009-1 12699877

[B24] Kallen C. B. Lazar M. A. (1996). Antidiabetic thiazolidinediones inhibit leptin (ob) gene expression in 3T3-L1 adipocytes. Proc. Natl. Acad. Sci. U.S.A. 93, 5793–5796 10.1073/pnas.93.12.5793 8650171 PMC39140

[B25] Kanie N. Matsumoto T. Kobayashi T. Kamata K. (2003). Relationship between peroxisome proliferator-activated receptors (PPAR alpha and PPAR gamma) and endothelium-dependent relaxation in streptozotocin-induced diabetic rats. Br. J. Pharmacol. 140, 23–32 10.1038/sj.bjp.0705414 12967931 PMC1574012

[B26] Ketsawatsomkron P. Lorca R. A. Keen H. L. Weatherford E. T. Liu X. (2012). PPARgamma regulates resistance vessel tone through a mechanism involving RGS5-mediated control of protein kinase C and BKCa channel activity. Circ. Res. 111, 1446–1458 10.1161/CIRCRESAHA.112.271577 22962432 PMC3494760

[B27] Ketsawatsomkron P. Pelham C. J. Groh S. Keen H. L. Faraci F. M. Sigmund C. D. (2010). Does peroxisome proliferator-activated receptor-gamma (PPAR gamma) protect from hypertension directly through effects in the vasculature? J. Biol. Chem. 285, 9311–9316 10.1074/jbc.R109.025031 20129921 PMC2843178

[B28] Kleinhenz J. M. Kleinhenz D. J. You S. Ritzenthaler J. D. Hansen J. M. Archer D. R. (2009). Disruption of endothelial peroxisome proliferator-activated receptor-gamma reduces vascular nitric oxide production. Am. J. Physiol. Heart Circ. Physiol. 297, H1647–H1654 10.1152/ajpheart.00148.2009 19666848 PMC2781385

[B29] Kubota N. Terauchi Y. Miki H. Tamemoto H. Yamauchi T. Komeda K. (1999). PPAR gamma mediates high-fat diet-induced adipocyte hypertrophy and insulin resistance. Mol. Cell 4, 597–609 10.1016/S1097-2765(00)80210-5 10549291

[B30] Kusinski L. C. Stanley J. L. Dilworth M. R. Hirt C. J. Andersson I. J. Renshall L. J. (2012). eNOS knockout mouse as a model of fetal growth restriction with an impaired uterine artery function and placental transport phenotype. Am. J. Physiol. Regul. Integr. Comp. Physiol. 303, R86–R93 10.1152/ajpregu.00600.2011 22552791

[B31] Law R. E. Goetze S. Xi X. P. Jackson S. Kawano Y. Demer L. (2000). Expression and function of PPARgamma in rat and human vascular smooth muscle cells. Circulation 101, 1311–1318 10.1161/01.CIR.101.11.1311 10725292

[B32] Leesnitzer L. M. Parks D. J. Bledsoe R. K. Cobb J. E. Collins J. L. Consler T. G. (2002). Functional consequences of cysteine modification in the ligand binding sites of peroxisome proliferator activated receptors by GW9662. Biochemistry 41, 6640–6650 10.1021/bi0159581 12022867

[B33] Liu Y. Bubolz A. H. Shi Y. Newman P. J. Newman D. K. Gutterman D. D. (2006). Peroxynitrite reduces the endothelium-derived hyperpolarizing factor component of coronary flow-mediated dilation in PECAM-1-knockout mice. Am. J. Physiol. Regul. Integr. Comp. Physiol. 290, R57–R65 10.1152/ajpregu.00424.2005 16166207

[B34] Marx N. Duez H. Fruchart J. C. Staels B. (2004). Peroxisome proliferator-activated receptors and atherogenesis: regulators of gene expression in vascular cells. Circ. Res. 94, 1168–1178 10.1161/01.RES.0000127122.22685.0A 15142970

[B35] Marx N. Walcher D. (2007). Vascular effects of PPARgamma activators - from bench to bedside. Prog. Lipid Res. 46, 283–296 10.1016/j.plipres.2007.05.003 17637478

[B36] Mateev S. Sillau A. H. Mouser R. McCullough R. E. White M. M. Young D. A. (2003). Chronic hypoxia opposes pregnancy-induced increase in uterine artery vasodilator response to flow. Am. J. Physiol. Heart Circ. Physiol. 284, H820–H829 12433660 10.1152/ajpheart.00701.2002

[B37] Matsumoto T. Kobayashi T. Kamata K. (2008). Relationships among ET-1, PPARgamma, oxidative stress and endothelial dysfunction in diabetic animals. J. Smooth Muscle Res. 44, 41–55 10.1540/jsmr.44.41 18552452

[B38] McCarthy F. P. Drewlo S. English F. A. Kingdom J. Johns E. J. Kenny L. C. (2011a). Evidence implicating peroxisome proliferator-activated receptor-{gamma} in the pathogenesis of preeclampsia. Hypertension 58, 882–887 10.1161/HYPERTENSIONAHA.111.179440 21931072

[B39] McCarthy F. P. Drewlo S. Kingdom J. Johns E. J. Walsh S. K. Kenny L. C. (2011b). Peroxisome proliferator-activated receptor-gamma as a potential therapeutic target in the treatment of preeclampsia. Hypertension 58, 280–286 10.1161/HYPERTENSIONAHA.111.172627 21690483

[B40] Moll W. (2003). Structure adaptation and blood flow control in the uterine arterial system after hemochorial placentation. Eur. J. Obstet. Gynecol. Reprod. Biol. 110(Suppl. 1), S19–S27 10.1016/S0301-2115(03)00169-6 12965087

[B41] Moncada S. Rees D. D. Schulz R. Palmer R. M. J. (1991). Development and mechanism of a specific supersensitivity to nitrovasodilators after inhibition of vascular nitric-oxide synthesis *in vivo*. Proc. Natl. Acad. Sci. U.S.A. 88, 2166–2170 10.1073/pnas.88.6.2166 1848694 PMC51190

[B42] Nicolakakis N. Hamel E. (2010). The Nuclear receptor ppargamma as a therapeutic target for cerebrovascular and brain dysfunction in Alzheimer's disease. Front. Aging Neurosci. 2:21 10.3389/fnagi.2010.00021 20725514 PMC2912024

[B43] Osol G. Mandala M. (2009). Maternal uterine vascular remodeling during pregnancy. Physiology 24, 58–71 10.1152/physiol.00033.2008 19196652 PMC2760472

[B44] Phillips J. K. Vance A. M. Raj R. S. Mandala M. Linder E. A. Gokina N. I. (2012). Impact of experimental diabetes on the maternal uterine vascular remodeling during rat pregnancy. Reprod. Sci. 19, 322–331 10.1177/1933719111424435 22383782 PMC3343150

[B45] Roberts J. (1998). Endothelial dysfunction in preeclampsia. Semin. Reprod. Endocrinol. 16, 5–15 10.1055/s-2007-1016248 9654603

[B46] Roberts J. M. Pearson G. Cutler J. Lindheimer M. (2003). Summary of the NHLBI working group on research on hypertension during pregnancy. Hypertension 41, 437–445 10.1161/01.HYP.0000054981.03589.E9 12623940

[B47] Sharma S. Barton J. Rafikov R. Aggarwal S. Kuo H. C. Oishi P. E. (2013). Chronic inhibition of PPAR-gamma signaling induces endothelial dysfunction in the juvenile lamb. Pulm. Pharmacol. Ther. 26, 271–280 10.1016/j.pupt.2012.12.004 23257346 PMC3872991

[B48] Sibai B. Dekker G. Kupferminc M. (2005). Pre-eclampsia. Lancet 365, 785–799 15733721 10.1016/S0140-6736(05)17987-2

[B49] Sigmund C. D. (2010). Endothelial and vascular muscle PPARgamma in arterial pressure regulation: lessons from genetic interference and deficiency. Hypertension 55, 437–444 10.1161/HYPERTENSIONAHA.109.144170 20038751 PMC2819308

[B50] Stanley J. L. Andersson I. J. Poudel R. Rueda-Clausen C. F. Sibley C. P. Davidge S. T. (2012). Sildenafil citrate rescues fetal growth in the catechol-O-methyl transferase knockout mouse model. Hypertension 59, 1021–1028 10.1161/HYPERTENSIONAHA.111.186270 22392899

[B51] Waite L. L. Louie R. E. Taylor R. N. (2005). Circulating activators of peroxisome proliferator-activated receptors are reduced in preeclamptic pregnancy. J. Clin. Endocrinol. Metab. 90, 620–626 10.1210/jc.2004-0849 15562025

[B52] Waite L. L. Person E. C. Zhou Y. Lim K. H. Scanlan T. S. Taylor R. N. (2000). Placental peroxisome proliferator-activated receptor-gamma is up-regulated by pregnancy serum. J. Clin. Endocrinol. Metab. 85, 3808–3814 10.1210/jc.85.10.3808 11061543

[B53] Wareing M. Myers J. E. O'Hara M. Baker P. N. (2005). Sildenafil citrate (Viagra) enhances vasodilatation in fetal growth restriction. J. Clin. Endocrinol. Metab. 90, 2550–2555 10.1210/jc.2004-1831 15713717

[B54] Wieser F. Waite L. Depoix C. Taylor R. N. (2008). PPAR action in human placental development and pregnancy and its complications. PPAR Res. 2008, 527048 10.1155/2008/527048 18288290 PMC2234270

[B55] Zimmermann P. Eirio V. Koskinen J. Kujansuu E. Ranta T. (1997). Doppler assessment of the uterine and uteroplacental circulation in the second trimester in pregnancies at high risk for pre-eclampsia and/or intrauterine growth retardation: comparison and correlation between different Doppler parameters. Ultrasound Obstet. Gynecol. 9, 330–338 10.1046/j.1469-0705.1997.09050330.x 9201877

